# Therapeutic Potential of Selected Probiotic Strains in a Murine Model of Ovalbumin-Induced Atopic Dermatitis and Asthma

**DOI:** 10.3390/ijms262010097

**Published:** 2025-10-16

**Authors:** Fang-Yu Zhang, Chi-Yu Yang, Jong-Shian Liou, Chien-Hsun Huang, Pei-Yu Lin, I-Jen Wang

**Affiliations:** 1 Animal Technology Research Center, Agriculture Technology Research Institute, Miaoli 35053, Taiwanchiyu@mail.atri.org.tw (C.-Y.Y.); 2 Bioresource Collection and Research Center (BCRC), Food Industry Research and Development Institute, Hsinchu 30062, Taiwanchh@firdi.org.tw (C.-H.H.); 3 Clinical Medicine, China Medical University, 77 Puhe Road, Shenbei New District, Shenyang 110122, China; 4 Department of Pediatrics, Taipei Hospital, Ministry of Health and Welfare, Taipei 11267, Taiwan; 5 School of Medicine, National Yang Ming Chiao Tung University, Taipei 300093, Taiwan; 6 College of Public Health, China Medical University, Taichung 40402, Taiwan; 7 National Institute of Environmental Health Sciences, National Health Research Institutes, Miaoli 35053, Taiwan

**Keywords:** atopic dermatitis, asthma, ovalbumin, probiotic

## Abstract

Atopic dermatitis (AD) and asthma are key manifestations of the atopic march, characterized by a progressive development of allergic diseases from early skin inflammation to later respiratory involvement. Emerging evidence highlights the role of gut microbiota in modulating immune responses. However, the therapeutic potential of specific probiotic strains in preventing or mitigating the atopic march remains underexplored. This study aimed to evaluate the immunomodulatory and therapeutic effects of selected probiotic strains in a murine model of ovalbumin (OVA)-induced AD and asthma. Mice received oral administration of *B. plebeiu*, *B. ovatus*, *F. duncaniae*, *F. taiwanense*, and *F. prausnitzii* for four weeks before being exposed to OVA to induce AD and, later, asthma. Skin reactions were assessed after OVA application, and asthma was induced via aerosolized OVA. Afterward, blood and lung fluid samples were collected to evaluate immune markers such as total IgE, OVA-specific IgE, and IL-4. The results showed that *B. plebeius* improved skin histology in AD, while *B. ovatus* initially induced AD symptoms but later reduced them significantly between days 40 and 54. *B. plebeius* and *B. ovatus* reduced serum total IgE in asthma. *B. plebeiu*, *B. ovatus*, *F. duncaniae*, *F. taiwanense*, and *F. prausnitzii* significantly lowered OVA-IgE levels in serum and IL-4 levels in lung fluid (*p* < 0.05). These selected probiotic strains helped reduce allergic skin responses and, later, asthma by decreasing inflammation, particularly IL-4. These findings support the potential of these probiotics to prevent or mitigate the progression from AD to asthma and offer promising insight into targeted probiotic interventions for allergic diseases.

## 1. Introduction

Atopic dermatitis (AD) and asthma are chronic allergic diseases that have become increasingly prevalent worldwide, particularly among children. Both conditions involve complex immune dysregulation, impaired epithelial barrier function, and chronic inflammation. In recent years, accumulating evidence has highlighted the critical role of gut microbiota in shaping host immunity and maintaining systemic homeostasis. Dysbiosis—an imbalance in the gut microbial community—has been associated with heightened susceptibility to allergic diseases, including AD and asthma.

The etiology of atopic dermatitis (AD) development is complex and involves abnormal immune and inflammatory responses, including skin barrier defects, exposure to environmental factors, and neuropsychological factors [1,2,3,4]. About 70~80% of patients with AD present with external forms of AD and an increased serum immunoglobulin E (IgE) level [5]. Scratching from intense itching leads to skin damage, which leads to the release of cytokines and chemokines and, consequently, a further increase in skin permeability. This then further promotes the entry of allergens into the skin, which is also a cause of AD [6].

The use of animal models to simulate human AD patterns has greatly expanded our understanding of the disease and allowed for in-depth studies of its pathogenesis. Mice, dogs, and guinea pigs can develop symptoms similar to AD; however, mouse models are mainly used because they are easy to establish and maintain, are low cost, and most importantly, the genetic strain can be manipulated. Since the Nc/Nga mouse was first described as a spontaneously occurring AD model in 1997 [7], many mouse models have been developed. These animal models can be divided into three groups: (1) models induced by epidermal epicutaneous sensitizers; (2) transgenic mice selected to overexpress or lack certain genes; and (3) mice with spontaneously occurring AD-like skin lesions. These models display many features of human AD, allowing for a better understanding of the pathogenesis and treatment of the disease. These models are based on skin injury and allergen sensitization AD, which is AD induced by repeated epidermal sensitization of depilated skin with ovalbumin (OVA), and have been used in five mouse strains so far, including BALB/c and C57BL/6 mice [8]. Epidermally sensitized mice show increased scratching behavior and skin lesions characterized by epidermal and dermal thickening, CD4+ T-cell and eosinophil infiltration, and Th2 cytokines IL-4, IL-5, and IL-13. In addition, increased expressions of OVA-specific IgG1, IgE, and IgG2a in serum have been reported, with higher expressions of IL-4, IL-5, IL-13, and IFN-γ in the spleen cells of OVA-sensitized mice. In addition, OVA-sensitized mice have been reported to show increased asthmatic hyperresponsiveness after aerosol administration of OVA, a feature similar to that of most patients with AD [9,10].

Despite growing interest in the gut–skin–lung axis, the relationship between specific bacterial strains and allergic disease remains underexplored. Notably, the roles of *Bacteroides plebeius*, *Bacteroides ovatus*, *Faecalibacterium duncaniae*, *Faecalibacterium taiwanense*, and *Faecalibacterium prausnitzii* in the pathogenesis of AD and asthma are not well understood. Some studies suggest that there are lower levels of *B. plebeius* in children with asthma and reduced *F. prausnitzii* in patients with AD [11]. *F. duncaniae*, a recently reclassified butyrate-producing species, has shown anti-inflammatory activity in murine asthma models [12], yet human data remain limited. The immunoregulatory potential of *B. ovatus* has been suggested in early-life microbiome studies [13], while *F. taiwanense*, a novel species isolated in Taiwan, remains largely uncharacterized in allergic disease. *B. plebeius*, originally identified in Japanese individuals with seaweed-rich diets, can degrade porphyran and may influence mucosal immunity [14]. *B. ovatus*, a common gut commensal, supports IgA production and gut barrier function. *F. duncaniae* and *F. prausnitzii* are key producers of butyrate, which strengthens epithelial integrity and promotes anti-inflammatory regulatory T-cell responses [15]. 

Given the limited number of comprehensive studies on this topic, we aim to investigate the associations and underlying mechanisms of these strains in the context of AD and asthma. By elucidating their immunomodulatory effects, we hope to contribute to the development of microbiome-based therapeutic strategies for allergic diseases and the atopic march.

## 2. Results

### 2.1. Body Weight Change

**Figure 1:**
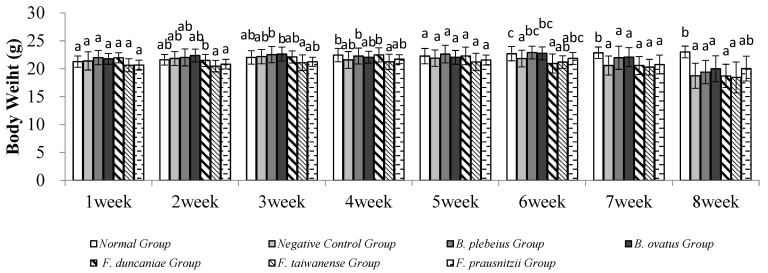
Weight changes. a–c There was no significant difference (*p* > 0.05) between the same letters.

No animals died during the experiment. There was no significant difference in body weight among the groups in the first week (*p* > 0.05). In the second week, the body weight of the *B. ovatus* group was significantly higher than those of the *F. taiwanense* and *F. prausnitzii* groups (*p* < 0.05). In the third week, the body weights of the *B. plebeius* and *B. ovatus* groups were significantly higher than that of the *F. prausnitzii* group (*p* < 0.05). In the fourth week, the body weights of the normal group and the *B. plebeius* group were significantly higher than that of the *F. taiwanense* group (*p* < 0.05). There was no significant difference among the groups in the fifth week. In the sixth to eighth weeks, the body weight of the normal group was significantly higher than those of the other groups (*p* < 0.05) (Figure 1).

### 2.2. Total Serum IgE

**Figure 2:**
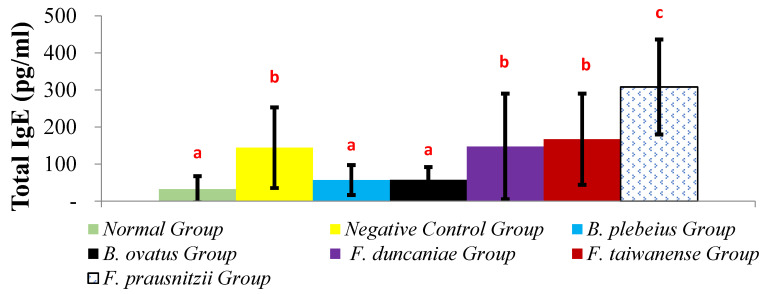
Total IgE content in serum. a, b, and c represent significant differences (*p* < 0.05).

The serum IgE levels in the *B. plebeius* and *B. ovatus* groups were 57.01 ± 40.36 pg/mL and 57.75 ± 34.40 pg/mL, respectively, which were significantly lower than that in the negative control group. The *F. duncaniae*, *F. taiwanense*, and *F. prausnitzii* group serum IgE levels were 147.56 ± 142.51, 167.24 ± 122.97, and 308.18 ± 128.24 pg/mL, respectively. Except for the *F. prausnitzii* group, no other groups showed statistically significant differences from the negative control group. There was a significant difference between the oral *B. ovatus* and *B. plebeius* group (*p* < 0.05) and the negative control group (Figure 2).

### 2.3. OVA-IgE Content in Serum

**Figure 3:**
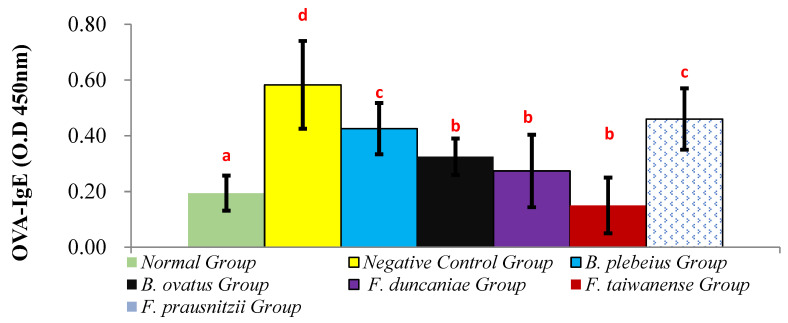
OVA-IgE content in serum. a, b, c, and d represent significant differences (*p* < 0.05).

The expression levels of OVA-IgE in the serum of the normal group and the negative control group were 0.19 ± 0.06 and 0.58 ± 0.16, respectively, and the expression level of OVA-IgE in the normal group was significantly lower than that in the negative control group (*p* < 0.001). In addition, the serum levels of OVA-IgE in the *B. plebeiu*, *B. ovatus*, *F. duncaniae*, *F. taiwanense*, and *F. prausnitzii* groups were 0.43 ± 0.09, 0.33 ± 0.07, 0.27 ± 0.13, 0.15 ± 0.10, and 0.46 ± 0.11, respectively. The OVA-IgE expression of the *B. plebeiu*, *F. prausnitzii*, *B. ovatus*, *F. duncaniae*, and *F. taiwanense* treatments displayed a downward trend from the negative control value (Figure 3). 

### 2.4. Expression of IL-4 in Alveolar Flushing Fluid

**Figure 4:**
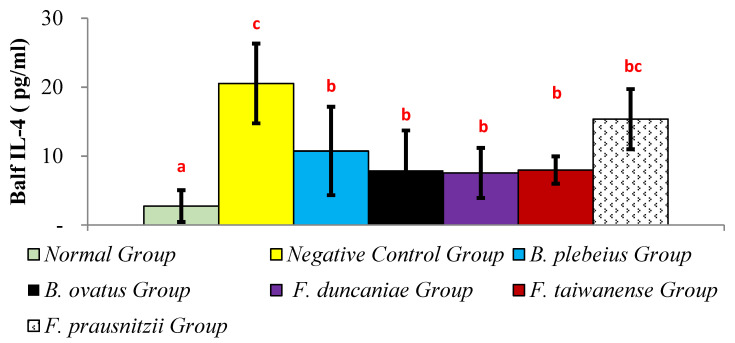
IL-4 contents in lung flushing fluid. a, b, and c represent significant differences (*p* < 0.05).

The level of IL-4 in the alveolar lavage fluid of the normal group was significantly lower than that in the negative control group (2.75 ± 2.31 pg/mL vs. 20.54 ± 5.78 pg/mL; *p* < 0.05). In addition, the IL-4 levels in the alveolar lavage fluid of the *B. plebeius*, *B. ovatus*, *F. duncaniae*, *F. taiwanense*, and *F. prausnitzii* groups were 10.74 ± 6.41, 7.92 ± 5.79, 7.56 ± 3.63, 7.98 ± 1.98, and 15.36 ± 4.36 pg/mL, respectively, all of which were significantly lower than those in the negative control group (*p* < 0.05) (Figure 4).

### 2.5. Expression of IFN-γ in Alveolar Flushing Fluid

**Figure 5:**
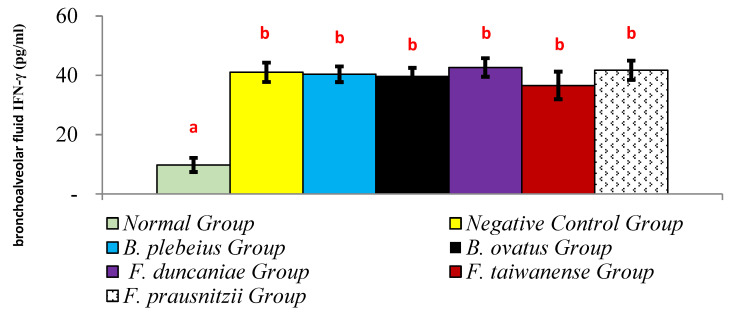
Expression of IFN-γ in alveolar flushing fluid. a and b represent significant differences (*p* < 0.05).

The level of IFN-γ in the alveolar flushing fluid in the normal group was significantly lower than that in the negative control group (9.77 ± 2.39 pg/mL vs. 41.02 ± 3.26 pg/mL; *p* < 0.05). In addition, the levels of IFN-γ in the alveolar washing fluid of the *B. plebeiu*, *B. ovatus*, *F. duncaniae*, *F. taiwanense*, and *F. prausnitzii* groups were 40.35 ± 2.66, 39.84 ± 2.69, 42.65 ± 3.13, 36.58 ± 4.65, and 41.69 ± 3.26 pg/mL, respectively, and there was no significant difference compared with the negative control group (*p* > 0.05) (Figure 5).

The results showed that, except for Group F, all probiotic groups exhibited a significant decrease in IL-4 compared with the negative control group. IL-4 primarily acts through the Th2 pathway, as it can drive and induce Th2 helper cells, whose main function is to mediate immune responses against extracellular multicellular parasites. It is therefore not strictly necessary to measure the number and activity of Th2 cells in order to demonstrate that these immune responses are related to Th2.

### 2.6. Skin Condition Observation

**Figure 6:**
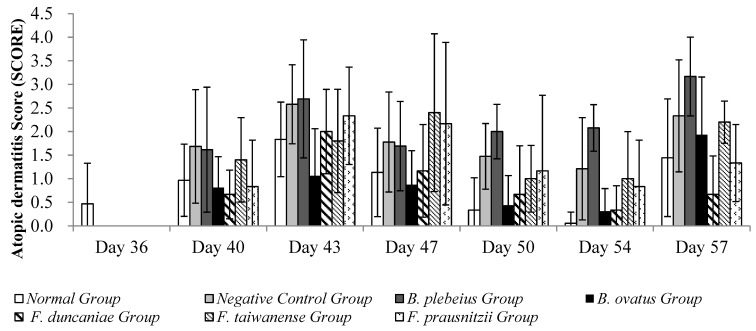
Skin condition observation.

The observation results of the skin condition of each group in the stimulation-induced AD animal model with OVA are summarized in Figure 6. During the induction period, observations and recordings were made once at intervals of 3 to 4 days, and skin stimulation was performed twice in total. During the first skin stimulation period (days 40~43), the average AD score examination at the stimulation site in the negative control group was significantly higher than that of the normal group (*p* < 0.05) (Figure 6). Compared with the negative control group, the average skin SCORE in the *B. ovatus* group was significantly lower (*p* < 0.05), while the average skin SCORE in the *B. plebeius* group was lower but without significance (*p* > 0.05).

When the animals were unwrapped and not in contact with the allergen (OVA), the skin condition of the animals in each group gradually returned to normal. On days 47 and 50, the average SCORE of the stimulated skin were 1.3 and 0.5 in the normal group; 2.5 and 1.8 in the negative control group; 0.88 and 0.44 in the *B. plebeius* group; and1.17 and 0.67 in the *F. duncaniae* group. The average skin SCORE in the negative control group was significantly higher than the scores in the normal group and *B. ovatus* group (*p* < 0.05), while the average score in the *B. ovatus* group was close to that of the normal group.

During the second skin stimulation induction with OVA (days 54 and 57), the severity of dermatitis in the control group was lower than that of the first stimulation induction, and the recovery time was shortened. The *B. ovatus* and *F. duncaniae* group scores were 0.31, 1.93 and 0.33, 0.67, respectively. In addition, the average scores in the negative control group were higher than those in the other groups, and there was a significant difference with the normal group (*p* < 0.05).

## 3. Discussion

This study presents an interesting and unique approach to understanding the role of probiotics in AD and the development of AD. Not many studies have utilized this specific investigation method, which combines oral probiotic administration with OVA skin stimulation to create a model that mirrors the progression of AD to asthma. In this study, the AD model was induced by OVA skin stimulation following 4 weeks of oral administration of probiotics (*B. plebeius*, *B. ovatus*, *F. duncaniae*, *F. taiwanense*, and *F. prausnitzii*) and OVA. The oral route of administration was chosen to simulate the most common mode of probiotic intake in humans. OVA was selected, as it is widely recognized in immunological research as a standard immunogen capable of inducing robust antigen-specific immune responses in animal models; this aligns with the pathophysiology of atopic dermatitis, which is closely linked to allergic immune reactions against environmental antigens. Furthermore, AD is closely associated with other allergic diseases and is considered the initial step of the so-called “atopic march,” with nearly 80% of patients subsequently developing asthma or allergic rhinitis. During the final 3 days of the test, a 3% OVA aerosol was administered to induce the asthma model. The expression of IgE in the serum, cytokine levels in the lung lavage fluid, and the condition of the skin at the stimulation site were then evaluated.

When an allergic reaction occurred, Th2 cells secreted IL-4, which increased the concentration of IgE antibodies in the serum. Our findings are similar to those of a previous study, in which the linear discriminant analysis effect size indicated that Bacteroidaceae and Porphyromonadaceae could act as possible biomarkers associated with the diagnosis of AD. Probiotics can modify the composition of the gut microbiome, which may have an impact on the incidence and development AD. Thus, it can be concluded that the development of AD is significantly influenced by gut microbiota [8]. 

AD is a chronic, relapsing inflammatory skin disease, whose pathogenesis has not been fully understood. Although some AD mouse models already exist, it is not easy to establish a model that can represent the natural development of human AD [10]. In this study, we developed an AD model based on the inside–outside theory and investigated the effects of *B. plebeius*, *B. ovatus*, *F. duncaniae*, *F. taiwanense*, and *F. prausnitzii*. Probiotics have been considered immunomodulators in allergic diseases. The AD model resulted in skin erythema and itching and increased skin inflammation, as assessed by mouse skin scoring. Oral administration of *F. duncaniae* and *F. prausnitzii* alleviated all the disease parameters mentioned above. In the AD model, OVA-specific IgE and total IgE were expressed, but these expressions were reduced in the AD mice treated with *B. plebeius*, *B. ovatus*, *F. duncaniae*, and *F. taiwanense*. According to Asahi data, atopic manifestations occur in a series, usually with AD in infancy and allergic rhinitis and/or asthma later in life. In this study, 3% OVA was administered into the trachea after the experiment to induce asthma. The late-stage atopic dermatitis–asthmatic pattern is defined as immune dysregulation driven by Th2 cells, characterized by elevated levels of interleukin (IL)-4, IL-13, and IL-5 [9]. Administration of *B. plebeius*, *B. ovatus*, *F. duncaniae*, *F. taiwanense*, and *F. prausnitzii* significantly reduced the level of IL-4 in lung lavage fluid.

## 4. Materials and Methods

### 4.1. Study Animals

Female BALB/c mice were purchased from the National Applied Research Laboratories (Tapei city, Taiwan) and were allowed to acclimate for at least one week before exposure. All the animal experiments and care were approved by the Institutional Animal Care and Use Committee (IACUC) of the Agricultural Technology Research Institute. Mice (weighing 19–22 g) had ad libitum access to rodent chow and water. The environment was set to a 12 h dark/light cycle, with a temperature of 24 ± 2 °C and 50 ± 20% relative humidity.

### 4.2. Study Design and Procedures

#### 4.2.1. OVA-Induced AD Animal Model

The animals were randomly divided into six groups (n = 12 in each group) in Table 1, in which the average weight of animals in each group should not exceed 20%: Group A (normal group), intraperitoneal injection of normal saline (0.2 mL/mouse) + saline (0.2 mL/mouse, oral administration); Group B (negative control group), intraperitoneal injection of OVA (20 μg/0.2 mL/mouse) + saline (0.2 mL/mouse, oral administration); Group C (*B. plebeius* group), intraperitoneal injection of OVA (20 μg/0.2 mL/mouse) + *B. plebeius* (0.2 mL/mouse, oral administration); Group D (*B. ovatus* group), intraperitoneal injection of OVA (20 μg/0.2 mL/mouse) + *B. ovatus* (0.2 mL/mouse, oral administration); Group E (*F. duncaniae* group), intraperitoneal injection of OVA (20 μg/0.2 mL/mouse) + *F. duncaniae* (0.2 mL/mouse, oral administration); Group F (*F. taiwanense* group), intraperitoneal injection of OVA (20 μg/0.2 mL/mouse) + *F. taiwanense* group (0.2 mL/mouse, oral administration); and Group G (*F. prausnitzii* group), intraperitoneal injection of OVA (20 μg/0.2 mL/mouse) + *F. prausnitzii* group (0.2 mL/mouse, oral administration).

The AD animal model was induced by OVA, which was purchased from Sigma-Aldrich A5503 (Darmstadt, Germany). Before local sensitization, the hair on the backs of the animals was shaved. OVA was applied to the skin surface, which was then bandaged and fixed for local stimulation. The application area was about 1 × 1 cm^2^. Systemic sensitization was performed by intraperitoneal injection of OVA (20 μg/200 μL/mouse) for 1 week, followed by stimulation of the skin with OVA (100 μg/100 μL/mouse) for 7 days. This constituted one cycle, and a total of three cycles were performed.

#### 4.2.2. OVA-Induced Late-Stage Asthma Animal Model

After the fourth week of the test period, as shown in the test flowchart, OVA (20 μg/0.2 mL/mouse, i.p.) was injected intraperitoneally every 2 weeks, and the asthma model was continuously induced with a 2% OVA aerosol for 3 days before sacrifice and continued every 2 weeks during the induction period. Feeding with probiotics was conducted once a day with a feeding needle. The serum levels of total IgE were measured by ELISA. The serum IgE level in the normal group was significantly lower than that in the negative control group (29.25 ± 16.84 ng/mL vs. 104.16 ± 67.95 ng/mL; *p* < 0.05). ELISA was performed using specimens from 72 animals, with all measurements taken at a wavelength of 450 nm. All assays were conducted with commercially available kits. ELISA was used to measure the expression level of OVA-IgE in serum and IL-4 in bronchoalveolar lavage fluid. The spectrophotometer used was TECAN Sunrise. The following reagents and kits were applied: Ovalbumin (Sigma, A5503, Darmstadt, Germany), Mouse Total IgE ELISA Kit (Invitrogen, 88-50460-88, Carlsbad, CA, USA), OVA-IgE ELISA Kit (BioLegend, LEGEND MAX™, San Diego, CA, USA), and Mouse IFN-γ ELISA Kit (Invitrogen, 88-7314-88, Waltham, MA, USA). The mice were challenged with aerosolized OVA (3%) for three consecutive days. The mice were exposed to OVA for sensitization and challenge to induce AD in the animal model, systemic sensitization was performed by intraperitoneal injection, and the normal animal group was injected with saline. Before local sensitization, the animal’s back hair was shaved, and then OVA was applied to the skin surface and then bandaged and fixed to induce local irritation. The smearing area was about 1 × 1 cm^2^. The normal animal group was smeared with saline. Systemic sensitization was performed by intraperitoneal injection of OVA (20 μg/200 μL/mice) and then stagnation for 1 week, and then OVA (100 μg/100 μL/mice) was used to stimulate the skin for 7 days. This was one cycle, with three cycles required in total.

**Figure 7:**
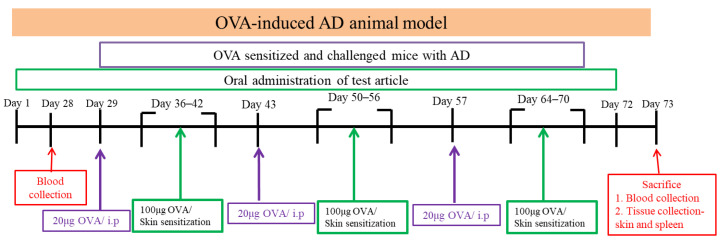
Flowchart of the animal experiment. Six groups of mice were orally administered probiotics or saline for 73 days continuously. The mice were intraperitoneally injected with OVA (20 μg) and Al(OH)3 in sterile saline on days 29, 43, and 57. The mice were sensitized with a patch containing 100 μg of OVA, applied to the shaved dorsal skin for 1 week per cycle. This procedure was repeated three times to induce the atopic dermatitis (AD) model. All animals in each group continued to the next experiment, in which they were challenged with aerosolized OVA (3%) for 3 days. The animals were sacrificed 24 h after the last OVA exposure to obtain bronchoalveolar fluid and lung tissue. OVA = ovalbumin, i.p. = intraperitoneal. Skin condition observation.

The flowchart is presented below (Figure 7).

After stimulating the skin with OVA, we observed and evaluated the skin using an AD score examination every 3 to 4 days. After stimulating the skin with OVA, the skin condition was observed and scored, and pictures were taken once every 3 to 4 days. The AD score examination for the severity of dermatitis was assessed according to four symptoms: (1) erythema, (2) edema/papulation, (3) excoriation, and (4) lichenification. Each symptom was scored from 0 to 3 (none, 0; mild, 1; moderate, 2; severe, 3) [16].

#### 4.2.3. Blood Collection and Processing Methods

Before the test, blood was collected in blood collection tubes without anticoagulants using a lancet. After the test, Blood was collected from the cheek vein of mice using a lancet. We centrifuged the blood without anticoagulants at 4 °C at 3500 rpm for 10 min and then took the supernatant as serum and stored it at −40 °C until analysis.

#### 4.2.4. Lung Washing Fluid Collection

We used 0.5 mL Phosphate Buffered Saline (PBS) to lavage the lungs three times. In addition, we collected at least 1 mL of lung washing fluid, centrifuged it at 2000 rpm using an Eppendorf Centrifuge 5810R (Songjiang District, Shanghai, China) at 4 °C for 15 min, collected the supernatant, and stored it at −80 °C for the subsequent analysis of IL-4 and IFN-γ. The washed lungs were stored in 10% neutral formalin for subsequent analysis. ELISA (Invitrogen, USA) was used to measure the expression level of IFN-γ in the alveolar flushing fluid.

Within 24 h of the final challenge, we sacrificed the mice and collected blood samples. We collected the blood from the abdominal aorta, which we then centrifuged (3500 rpm at 4 °C for 15 min) and stored at −70 °C. We used ELISA kits to determine serum IgE concentrations.

We washed the lungs four times with 0.3 mL of PBS (1.2 mL). We centrifuged the bronchoalveolar fluid at 2000 rpm at 4 °C for 10 min. We centrifuged the supernatant of the bronchoalveolar fluid at 3000 rpm at 4 °C for 10 min, which we then stored at −70 °C. We used ELISA kits to determine the IL-4 and TNF-α concentrations in the bronchoalveolar fluid.

#### 4.2.5. Average Skin SCORE

We assigned the following scores to the groups using Atopic dermatitis score: No symptoms-0, Scratching—1 point, Redness and swelling—2 points, Skin damage and scaling—3 points, Mucus production—4 points, Pus and blood discharge—5 points [16].

### 4.3. Statistical Analysis

Experimental results are reported as mean ± standard deviation (SD). We used SPSS software version 22.0 (IBM, Armonk, NY, USA) for data processing. We performed statistical analysis using one-way ANOVA, followed by the Duncan’s *t*-test. We considered *p* < 0.05 to be statistically significant.

## 5. Conclusions

The results of this study reveal that probiotics, particularly *B. plebeius*, *B. ovatus*, *F. duncaniae*, and *F. taiwanense*, play an important role in alleviating symptoms of AD, including skin erythema, itching, and inflammation, as shown by improved mouse AD skin scoring. Additionally, these probiotics have been shown to reduce levels of OVA-specific IgE and total IgE, which are key markers of allergic reactions. This study also demonstrated that the immunopathophysiology of asthma and atopic dermatitis is heterogeneous, and not all patients exhibit Th2-driven inflammation. Likewise, IgE-dependent sensitization is not an obligatory requirement for the development of these diseases. We have revised the relevant statements in the manuscript to reflect this more nuanced understanding, citing the recent literature that highlights endotypic diversity in both asthma and atopic dermatitis. Notably, treatment with these probiotics led to a reduction in IL-4 levels in bronchoalveolar lavage fluid. These findings suggest that probiotic intervention can attenuate allergic immune responses, thereby improving both AD and its progression to asthma, and may offer a promising therapeutic strategy for managing allergic diseases.

## Figures and Tables

**Table 1:** Animal group.

Group	Oral Administration	Induced	Animals
A	normal group	-	12
B	negative control group	intraperitoneal injection of OVA	12
C	*B. plebeius* group		12
D	*B. ovatus* group		12
E	*F. duncaniae* group		12
F	*F. taiwanense* group		12
G	*F. prausnitzii* group		12

## Data Availability

The data presented in this study are available on request from the corresponding author due to privacy concerns and the sensitive nature of medical records.
